# Targeting undernutrition in Haiti: a spatial analysis for improving food security and reducing stunting in children under five

**DOI:** 10.1017/S1368980025101237

**Published:** 2025-10-06

**Authors:** Delia Atzori, Ben Sonneveld, Lia van Wesenbeeck

**Affiliations:** 1 Vrije Universiteit Amsterdam, Amsterdam Centre for World Food Studies (ACWFS), De Boelelaan 1105, Room 10A-64 (Secretariat Department of Economics), 1081 HV Amsterdam, The Netherlands; 2 Vrije Universiteit Amsterdam, Athena Institute, Faculty of Science, De Boelelaan 1105, Room 0E-20 (Secretariat Athena Institute), 1081 HV Amsterdam, The Netherlands; 3 Vrije Universiteit Amsterdam, School of Business and Economics, De Boelelaan 1105, School of Business and Economics, 1081 HV Amsterdam, The Netherlands

**Keywords:** Stunting, Spatial Analysis, Haiti, Food Policy

## Abstract

**Objective:**

This study aims to contribute to enhanced food security in Haiti through proposing targeted local interventions. Employing a spatially explicit tool, the research supports decision-making by relating undernutrition to socio-economic conditions and biophysical factors.

**Design:**

Georeferenced Demographic and Health Survey (DHS) conducted in 2016–2017 combined with spatial environmental information was used for a multivariate linear regression model to identify factors associated with stunting prevalence. Missing data were imputed through kernel density regression. We converted the structural relationship estimated for the territory of Haiti into a decision support tool by adding fixed effects at communal level. Various policy scenarios were analysed.

**Setting:**

Haiti, with spatial data across the 134 communes.

**Participants:**

The analysis included 5623 children under five and their mothers, sourced from DHS data.

**Results:**

Approximately 22 % of all children were stunted. Implementation of the LimitedIntervention development scenario led to a 2·5 % reduction in stunting, while the ModerateIntervention and FullIntervention scenarios achieved more significant reductions of 6 % and 10 %, respectively. Areas with highest stunting incidence benefit most from interventions.

**Conclusions:**

This tool supports decisionmakers by assessing the impact of interventions at commune level and selecting areas where interventions exert the most significant effects. The study suggests to apply a strategy that starts in relatively safe communes and then scales to other areas. The flexible approach adopted in this study allows applications in other countries or regions to assess the prevalence of undernutrition among children under five.

## Graphical abstract

**Figure:**
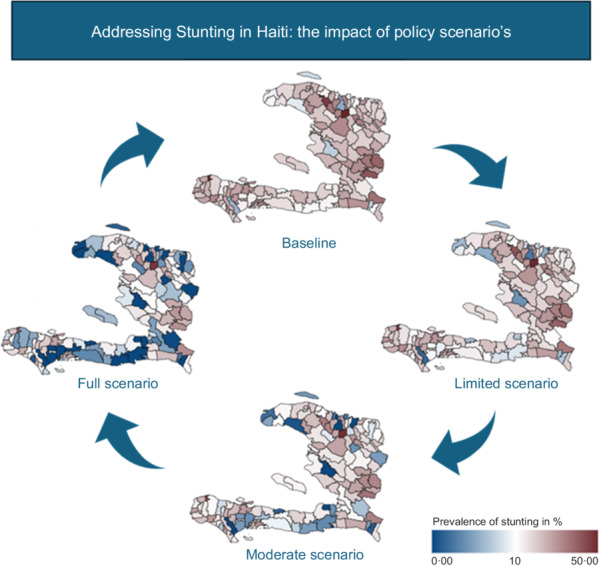

In April 2023, the UN highlighted the urgent need for assistance in Haiti as violent clashes between governmental forces and local gangs intensify^(1)^. Due to prevailing security issues, the island suffers from persistent economic stagnation and widespread poverty that seriously affect food security. The latest official poverty estimate in the Human Development Report^(2)^ shows that over 58·5 % of the Haitians lived below the national poverty line and 24·5 % fell below the extreme poverty line of USD 1·90 per day^(3)^. The prevalence of undernourishment went down from 51 % in 2001 to 43 % in 2016 but on the rise again with 47 % in 2020^(4)^. Sadly, the first victims of this stagnation are children. Under-five mortality stands at 57 per 1000 live births^(5)^, while 22 % of children under five are stunted and 4 % wasted^(4)^, compared with global rates of 37 per 1000 live births, 22·3 % and 6·8 %, respectively^(5)^. These numbers highlight a significant risk to Haiti’s future development, as malnutrition during childhood is known to have lasting impacts on mental and physical abilities. Additionally, the sad long-term structural physical and mental effects of stunting also take their toll in the loss of productive life years and related negative impact for the economy at national level. Galasso & Wagstaff^(6)^ found that in today’s workforce an average GDP per capita of 8 % in Haiti is lost due to childhood stunting.

The rise in food insecurity since 2009 is at par with Haiti’s declining agricultural output that no longer meets domestic demand, increasing the country’s dependency on (expensive) food imports. Hence, the cry for help in the UN press release can be interpreted as an appeal to increase the empowerment of the poor to curb negative trends, making them less dependent on erratic policies and volatile foreign aid and trade programs.

The current situation of Haiti cannot be understood without considering its history. Being the proud product of the slave led Haitian Revolution in 1791, the independent state of Haiti (1804) has ever since faced periods of authoritarian rule, military coups and political violence. France, the former colonising power, and the United States both have had a profound impact on the island’s political and economic development (‘repayments’ to France took up a large part of revenues in the first century of its existence^(7)^, while the US occupied Haiti from 1914 to 1934, and has influenced the political process ever since^(7)^). From 1957 to 1986, the country was under an autocratic regime, followed by a period of political instability that led to the establishment of a UN peacekeeping mission MINUSTAH. The combination of political violence and recurrent natural disasters has left Haiti in a state of vulnerability and fragility that hampered the country’s ability to implement long-term development strategies and invest in disaster preparedness and resilience of food systems^(8,9)^. An earthquake devastated the country in 2010; recovery efforts were harmed further when cholera started spreading along the Artibonite River. In 2016, Hurricane Matthew was the strongest recorded storm to hit Haiti since 1964. The impact on food security again was immense. Crops were destroyed just before harvest time, and the already fragile infrastructure was further damaged^(10)^.

Haiti shares important disadvantages with other Small Island Developing State (SIDS) in achieving Food and Nutrition Security. First, their remoteness from major markets, combined with their small size, leads to high transportation costs^(11)^ and high food prices, causing a shift of poor people’s daily food intake, replacing more nutritious and healthy food by lower quality meals^(12)^. Second, Haiti’s limited land area poses an inherent constraint on food production. This challenge is further exacerbated by unfavourable soil and terrain characteristics, which further reduce the land’s suitability for crop cultivation. For SIDS, Figure 1 maps the land quality ‘high’, ‘moderate’ and ‘low’ for high and low input conditions^(13)^ against the cultivated area^(14)^. Low input farming relies heavily on quality of natural resources, while under high input levels, the natural resource base is largely controlled and manipulated with agro-chemical, irrigation and machinery. The share of agricultural land is calculated for the year 2017. As can be seen, even under high-input conditions, moderately suited land is used as agricultural land, leading to low yields and further degradation of lands.

**Figure 1.**
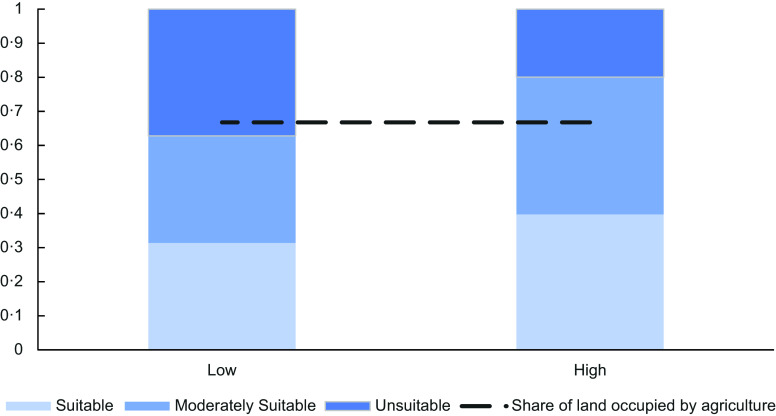
Land suitability classes for low and high input levels as share of total land against cultivated agricultural land for SIDS countries, year 2017^(13,14)^.

In contrast to many other SIDS, Haiti did not inherit large areas of cash crops from a colonial past. However, the terms of the 1983 Caribbean Basin Initiative, a US initiative that aimed for free trade between the US and Central American and Caribbean countries led to an influx of highly subsidised rice from the US and a re-orientation of Haitian agriculture to export crops^(15)^. Therefore, Haiti moved from being self-sufficient in rice in the early 1980s to being a large importer: currently, 80 % of rice now consumed in Haiti is imported^(16)^. In total, the value of imported food is 51 % that of the value of locally produced food^(17)^.

SIDS, including Haiti, currently encourage self-sufficiency in producing fresh and healthy foods. However, support is needed in formulating and targeting food policy interventions, as priority areas and factors contributing to food insecurity are not yet fully understood^(18)^.

This paper aims to provide support to national and international experts who assist food policy making in Haiti, and SIDS in general, by creating a spatial decision support tool to plan where and how food programs should be implemented. The study uses the prevalence of stunting among children under five as a proxy for food security as it reflects the chronic malnutrition situation in a household. The designed decision support tool will evaluate stunting prevalence in relation to spatially explicit variables, allowing for the identification of regions where interventions would have the greatest impact. This provides the government with actionable options to engage in food security efforts at a local level, even if nationwide implementation remains challenging. The paper also seeks to illustrate the adaptable nature of our approach, making it applicable in other regions or countries where existing spatial information can be utilised to predict the spatial prevalence of stunting among children under five and assess the impact of interventions.

Our approach, a GIS-based multicriteria decision analysis, has been successfully applied in various studies to evaluate the impact of policy interventions. Sonneveld *et al.* ^(19)^ created a GIS-based multicriteria decision analysis to evaluate the impact of home gardens on food security in urban and peri-urban areas. Kwaku Kyem^(20)^ applied the approach as a tool for intervening in disputes over access to natural resources. Sonneveld *et al.* ^(21)^ used a GIS-based multicriteria decision analysis model for a spatially explicit evaluation of the impact of climate change on crop production in Benin. Mayorga-Martinez^(22)^ used a spatial multicriteria decision analysis to design nutrition-sensitive agriculture policy interventions. Wesenbeeck *et al.* ^(23)^ use a GIS-based multicriteria decision analysis approach to evaluate the impact of climate change scenarios on foods security under vulnerable populations in Sub-Saharan Africa. Sema *et al.* ^(24)^ used a multicriteria decision analysis to explain childhood stunting in irrigated and non-irrigated Kebeles in northwest, Ethiopia. Saha *et al.* ^(25)^ use a spatial decision support tool to analyse the impact of risk factors on the SDG to reduce stunting among children under five in Bangladesh.

The spatial model in our study is based on a semi-parametric estimation, and the model presents results in choropleth maps and synoptic tables that are easily interpretable and can support the decision-making process of policymakers. Of course, given that at present, Haiti is facing political instability and social unrest and is dependent on outside forces to maintain some order in the country, designing food policies may not seem the most acute task for the government. However, it is vital that the country has a strategy ready for use if the security situation allows longer-term planning again, as ensuring livelihoods will contribute to maintaining stability in the country. In addition, the strategies explored in this paper can be used first in small, safe, parts of the country and gradually scaled to include a larger area.

## Methods and data

### Study area and source data

This study relies on the Global Administrative Areas Database^(26)^ to link socio-economic data to geographical variables. The analysis for Haiti is carried out at the level of the 134 *communes*, the fourth level in the administrative hierarchy, following the country, departments and arrondissements. The average population of a commune is 85 000, though sizes vary widely: the smallest commune has 4000 residents, while the largest, Port-au-Prince, has 926 900. Port-au-Prince is twice the size of the second-largest commune, Carrefour, which has 460 500 inhabitants^(27)^.

**Figure 2.**
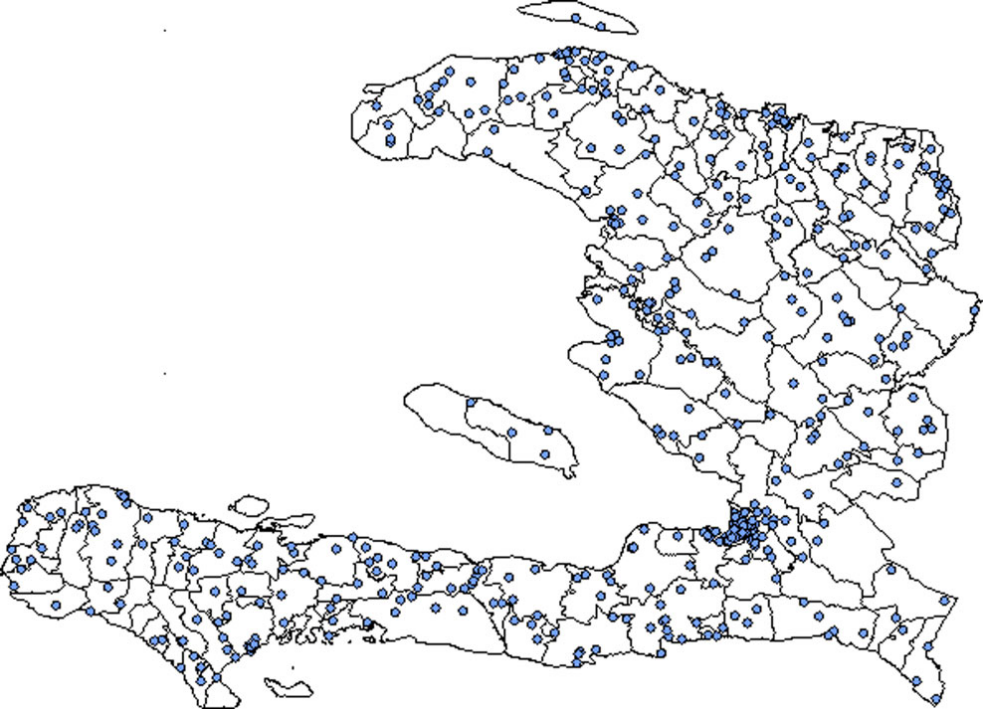
Administrative area at commune level with the location of DHS clusters.

Data on household characteristics were extracted from the 2016–2017 Haiti Demographic and Health Survey (DHS) Program^(28)^. DHS surveys are nationally representative and geo-referenced and include data on fertility, family planning, maternal and child health, gender, HIV/AIDS, malaria and nutrition. For reasons of privacy, georeferencing is done at the level of cluster, comprising about 25–30 households. Sample weights are used to ensure that the survey results are representative of the target population. The clusters are shown in Figure 2 on the commune map. Eleven communes, each with populations under 36 000, lack data altogether or do not report on stunting: Port-à-Piment, Chardonnières, Ⓘle-à-Vache, Saint-Jean du Sud, Pointe-à-Raquette, Chansolme, Vallières, Capotille, La Victoire, Chambellan and Cerca Carvajal.

### Variables used in this study

Below we introduce the dependent (2·2·1) and independent (2·2·2) variables that were used in this study.

#### Dependent variable

Stunting prevalence was selected as dependent variable to address the structural malnutrition status of the Haitian population, following the WHO definition that stunting ‘is the impaired growth and development that children experience from poor nutrition, repeated infection and inadequate psychosocial stimulation’. Stunting prevalence can be measured accurately and does not depend on perceptions of interviewees. Following international conventions, a child was labelled stunted if the height-for-age score was more than two standard deviations below the WHO Child Growth Standards mean. A total of 5623 children under five, from 4238 households, were included in the analysis.

#### Explanatory variables for modelling

In total, twenty-two explanatory variables were considered for the prediction model (Table 1). The variables were divided into three groups: maternal and child conditions, Water, Sanitation and Hygiene (WASH) conditions and food and agricultural variables.

**Table 1.** Independent variables selected for the analysis

Maternal and child conditions	WASH conditions	Food and nutrition security and agricultural aspects
Wealth index	Improved or unimproved toilet facility	Soil suitability
Education mother	Improved or unimproved drinking water source	Rainfall
Distance to healthcare facility (women)	Handwash facility (basic, limited or no facility)	Rural/urban environment
BMI mother	Time to obtain drinking water	Owns land usable for agriculture
Maternal height	Diarrhoea in last 2 weeks (child)	Minimum dietary diversity
Exclusively breastfed babies under 6 months		Dietary diversity (average food groups available)
Average months breastfeeding (whether or not exclusively)		
Birthweight		
Sex of child		
Children under five sleeping under a mosquito net		
Child received basic vaccination		

##### Maternal and child conditions

It is well- known that children from households with **higher wealth classes** are less likely to be stunted^(29,30)^. The same studies show that **low levels of maternal education** increase the likelihood of a child being stunted. Another important factor to consider is **breast-feeding**. Exclusively breastfed babies under 6 months of age have a reduced probability of being stunted^(30)^, although Ali *et al.* ^(31)^ found that prolonged breast-feeding was significantly related with (severe) stunting, probably the child is insufficiently introduced to other types of food. Other maternal factors are the **BMI** and **height of the mother**. Higher maternal height is associated with a lower risk of stunting^(31)^, and a lower BMI rate of the mother is associated with a higher risk on stunting^(32)^. Furthermore, Shahid *et al.* ^(33)^ found that stunting rates increase if the nearest **healthcare facility** is further away.

Child conditions are **birthweight, sex, mosquito net use and vaccination**. Ali *et al.* ^(31)^ found that males were more likely to be stunted, whereas Kang *et al.* ^(29)^ found that females were more likely to be stunted. A low birthweight is associated with higher odds of stunting^(31)^. Mosquito-borne diseases are common in Haiti, including the endemicity of malaria and dengue^(34)^. Mesfin *et al.* ^(35)^ found that the use of mosquito nets was associated with lower risk of stunting. Having up-to-date vaccinations was associated with a reduced risk to be stunted^(36)^.

##### Water, Sanitation and Hygiene conditions

Several studies considered holistic WASH programmes, including improving water quality, sanitation coverage and handwashing practices. Prüss-Ustün *et al.* ^(37)^ show that **WASH programmes** could positively influence children’s nutritional status, while Danaei *et al.* ^(38)^ identified **diarrhoea and a lack of improved sanitation** as the two main risk factors for stunting. Multiple studies associate specific WASH interventions, such as improved sanitation coverage or improved water sources with reduced stunting rates^(29–31)^. Finally, Sahiledengle *et al.* ^(39)^ found an association between **time to obtain drinking water** and stunting. Although this association was not statistically significant when corrected for potential confounders, we still included the variable in our gross list.

##### Food and nutrition security and agricultural aspects

In the literature, stunting prevalence was found to be higher in **rural areas** than in urban ones, although the difference is not always statistically significant^(29)^. Additionally, **owning land** suitable for crop cultivation shows a protective effect on stunting^(29,36)^. The **importance of diet** for stunting has been researched for decades. A cluster-randomised trial of Humphrey *et al.* ^(40)^ found a 21 % reduction of stunting prevalence as a result of a mixed-approach diet intervention. Other research supports the statement that inadequate diet contributes to stunting^(29,38)^. To cover agricultural conditions, we took **annual rainfall** as a proxy for water availability for crop and livestock^(41)^, while **soil suitability**, based on soil attribute data contained in the Harmonized World Soil Database (version 1), is shown to be closely related to food supply^(42)^. Of course, increased food supply does not immediately imply increased or more diverse food intake, but it can clearly contribute to it.

### Statistical analysis

Statistical analysis was conducted in SAS 9.4^(43)^. Figures and tables were created in Microsoft Excel and Power BI Pro. Descriptive statistics are summarised for categorical variables with frequency numbers and percentages, continuous variables are described by mean, sd, minimum and maximum (Results section). For the prediction model, the average per commune was calculated for continuous variables and the share for dichotomous variables.

The literature does not provide clear guidelines for the functional relationship between stunting prevalence and the variables that seem to be relevant. Hence, we follow usual conventions and assume a linear relationship between the independent variables and stunting prevalence. Under this assumption, we use a stepwise linear regression to identify independent variables that significantly contributed to the prediction of stunting prevalence, where we take a lenient threshold of significance (*P* < 0·1). Tests for multicollinearity were performed as well. Since we are fully aware that the linearity assumption is a very strong one that cannot be tested directly, we perform additional robustness tests by resampling. In addition, tests for normality were conducted using Shapiro–Wilk and Kolmogorov–Smirnov statistics and visualisations.

Formally, the model now is 
[1]

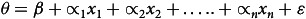




where 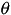 represents stunting prevalence and the vectors 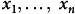 are the independent variables, with parameter vectors 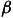 and 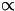 to be estimated. 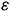 is the error term. A backward stepwise regression gradually eliminated variables to find a reduced model that has the optimal balance between power of explanation and simplicity. Second, a fixed effect at commune level (subscript *i*) was added, to represent possible impact of unobserved variables specific to the commune, that is, whether or not the commune has been targeted by interventions by donors or the government, the degree to which the commune has been affected by unrest and/or violence, and, associated to this, the level of cohesion and trust within the commune. It is well known that these factors can have a profound impact on children’s development^(44)^, but our dataset does not allow further exploration of these factors, hence our approach to model commune impact as a fixed effect 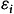 .

#### Ten-fold cross validation

The model [1] was tested for its sensitivity to inclusion or exclusion of observations and stability of the parameter estimations by a 10-fold cross-validation procedure^(45)^. In this procedure, the dataset is subdivided at random in ten sets of about equal size, and the model is estimated each time with nine subsets of the data. The resulting ten different parameter estimates are compared for their stability.

After estimation, the model is used as a prediction tool, by bounding the stunting prevalence between 0 and 1, leading to predicted stunting prevalence at commune level as 
[2]

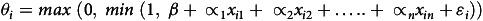




#### Imputation of missing observations

To apply the model in every commune, we had to impute missing values for variables selected in the regression analysis. For this, we needed to identify communes that are similar to communes for which observations are missing. Hence, a distance measure between communes was specified, using a kernel density estimation to assess the weighted average of different ‘neighbouring’ communes. After considering various variables that could be used as distance measure, we concluded that the simple Euclidian distance was most reliable to impute the missing data. The fact that the commune borders are purely administrative and do not respect natural borders (rivers, mountains) supports in our view the use of the Euclidian distance for imputation. In analogy with the previous example, as geological and agro-ecological variables show a continuous change when moving from one commune to the next, we follow Tobler’s first law of geography and assume that physical proximity will also result in more general similarity. For the distance function, we use the normal distribution equation that gives higher weights to observations of nearby communes. We acknowledge that we lack a more effective imputation method, but assert that there is no clear evidence of other dominant variables that would be better suited to construct a similarity measure.

## Results

### Descriptive statistics

Table 2 shows descriptive statistics for the variables included in the analysis. About 22 % of children under five is stunted. The majority of children belong to the poorest wealth index, 76 % does not sleep under mosquito nets, while only 19 % of children are fully vaccinated. WASH conditions are poor. Only 26 % of households avail of improved sanitation facilities, 53 % use improved drinking water sources almost 70 % have no handwash facility and on average there is a 28 min walking distance to obtain clean water. The study population lives largely in rural areas (69 %), and 67 % has access to land for agriculture. A minimum food diversity is only reached by 11 %. On average, a diet diversity of three food groups is observed. We conclude that the Haitian population is predominantly rural, mired in poverty, has poor healthcare for children, insufficient WASH conditions and a low diet diversity.

**Table 2.** Overall sample characteristics

Variable	Descriptive statistics				
	*n*	%	Mean	sd	Min–Max
Stunting					
Yes	1211	21·5			
No	4412	78·5			
Wealth index					
1 = poorest	1197	28·2			
2	953	22·5			
3	853	20·1			
4	709	16·7			
5 = richest	526	12·4			
Education mother					
No education	860	19·0			
Primary	1797	39·7			
Secondary	1721	38·0			
Higher	148	3·3			
Distance to healthcare facility, women					
No problem or not a big problem	2052	45·3			
Big problem	2474	54·7			
BMI mother in kg/m^2^			23·9	4·7	13·6–47·9
Maternal height in cm			159·3	6·3	102·0–182·4
Exclusively breastfed babies under 6 months					
Yes	267	38·9			
No	419	61·1			
Average months breastfeeding, whether or not exclusively			9·2	6·8	0–59
Birthweight			3182·0	952·3	500–6000
Sex of child					
Male	2832	50·4			
Female	2791	49·6			
Children under five sleeping under a mosquito net					
Yes	1324	23·6			
No	4299	76·4			
Child received full basic vaccination					
Yes	1070	19·0			
No	4553	81·0			
Diarrhoea in last 2 weeks, child					
Yes	1176	20·9			
No	4447	79·1			
Type of sanitation facility					
Improved	1081	25·5			
Unimproved	3157	74·5			
Drinking water source					
Improved	1617	53·3			
Unimproved	1418	46·7			
Handwash facility					
No facility	2940	69·4			
Limited, handwash facility on premises without soap and water	492	11·6			
Basic, handwash facility on premises with soap and water	806	19·0			
Time in minutes to obtain drinking water			28·1	35·2	0–360
Soil suitability, nine classes (1 = very suitable 9 = unsuitable)			3·2	1·5	1–9
Rainfall in mm			1021·4	127·3	763–1239
Rural/urban environment					
Urban	1305	30·8			
Rural	2933	69·2			
Owns land usable for agriculture					
Yes	2824	66·6			
No	1414	33·4			
Minimum dietary diversity, threshold 5/8 food groups available					
Yes	350	10·9			
No	2852	89·1			
Dietary diversity, average food groups available			2·5	1·6	0–8

### Explaining stunting prevalence

Variables presented in 3·1 were used for a stepwise regression analysis. The model fit presented by the R-square was 0·55, hence 55 % of the variation of stunting prevalence can be explained by the model. Our variance inflation factor fluctuates between 1 and 2·5 with a mean of 1·5, and hence no variables needed to be excluded because of multicollinearity. Tests for normality of the error term of the weighted sample in the regression analysis yielded contradicting conclusions. The Shapiro–Wilk test rejects the null hypothesis and assumes a non-normal distribution, while the Kolmogorov–Smirnov test fails to reject this suggesting a normal distribution. However, visualisation through Q-Q plots and histograms confirmed the assumption of normality.

Within the selected variables, we concentrate on standardised parameter estimates (SPE) which facilitates a comparison between individual variables and their impact on stunting rate (Table 3). Maternal education, well known for directly impacting earning potential and access to required resources and knowledge benefitting maternal care, has the largest effect. Second is sanitation facility (SPE: –0·30) indicating that improved sanitation reduces stunting; an important policy finding. Maternal height is also statistically significant in this combination of variables with a negative sign (SPE: –0·28). Exclusive breast-feeding has a negative effect, possibly because the child does not benefit from a more diverse diet, while it is undisputed that breast-feeding is beneficial for the child in the first months, after that, other foods need to be added to the child’s diet.

**Table 3.** Parameter estimates, se, t-value, *P*-value and standardise estimate

		R-Square 0·55		Adjusted R-square 0·51	
Variable	Parameter estimate	se	t-value	*P*-value	Standardised estimate
Intercept	239·96	78·40	3·1	0·00	0
Exclusive breastfeeding	0·07	0·03	2·1	0·04	0·23
Education mother	−0·23	0·08	−2·8	0·01	−0·35
Average height mother	−0·13	0·05	−2·5	0·02	−0·28
Sanitation facility	−0·16	0·06	−2·5	0·02	−0·30

**Figure 3.**
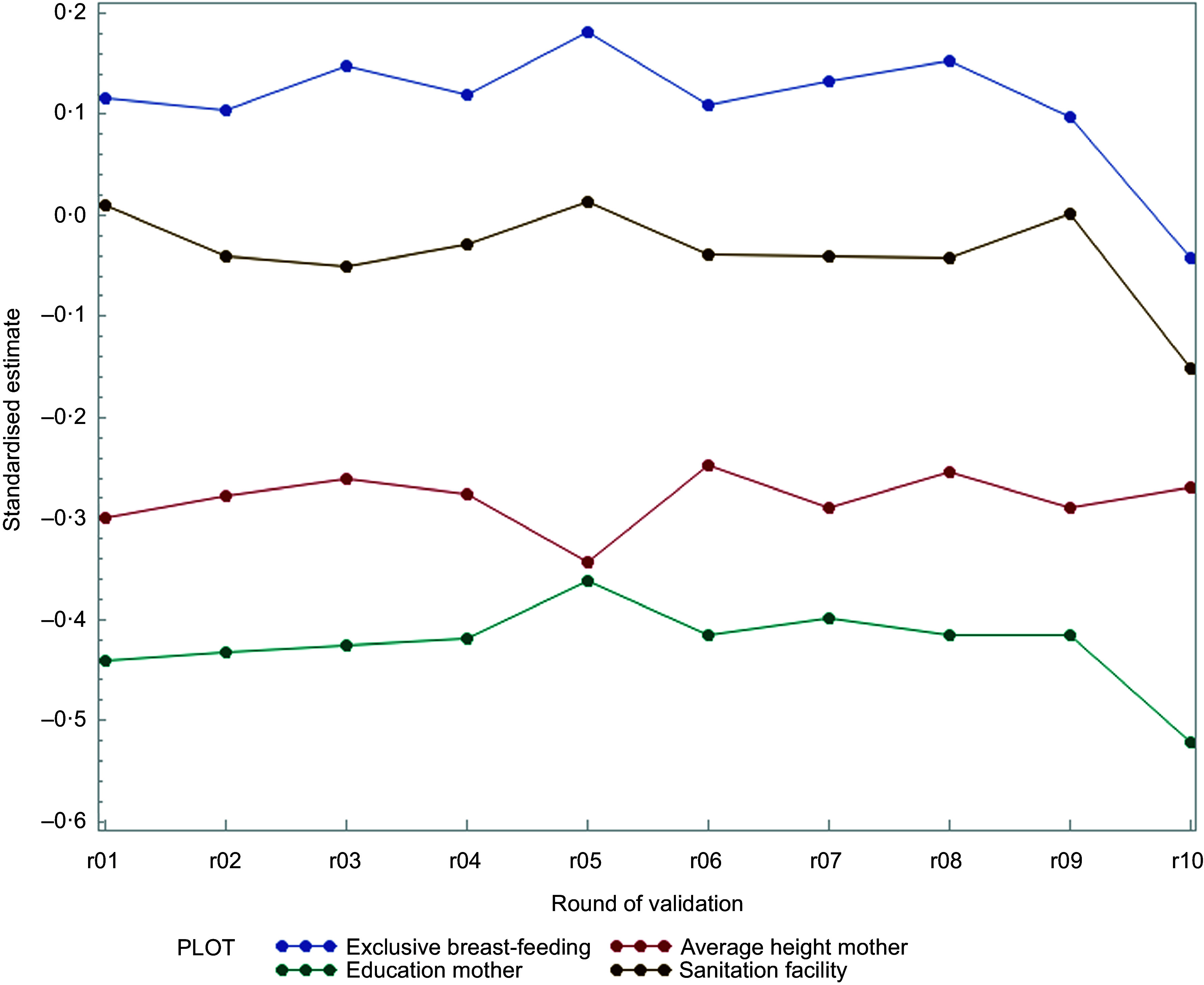
Results of ten-fold cross validation; standardised parameter estimates against round of estimation.

A 10-fold cross validation was conducted to test stability of estimated parameters for inclusion and exclusion of observations. From the ten models that were based on a subset of 90 % of the observations, all four variables were included in these ten models and no new variables were introduced. Figure 3 shows the standardised parameter estimates where only minor fluctuations are observed over the ten estimated rounds confirming that the estimated model will give stable outcomes, a property that we will use to convert the estimated model with the variables in Table 3 into a decision support tool.

**Figure 4.**
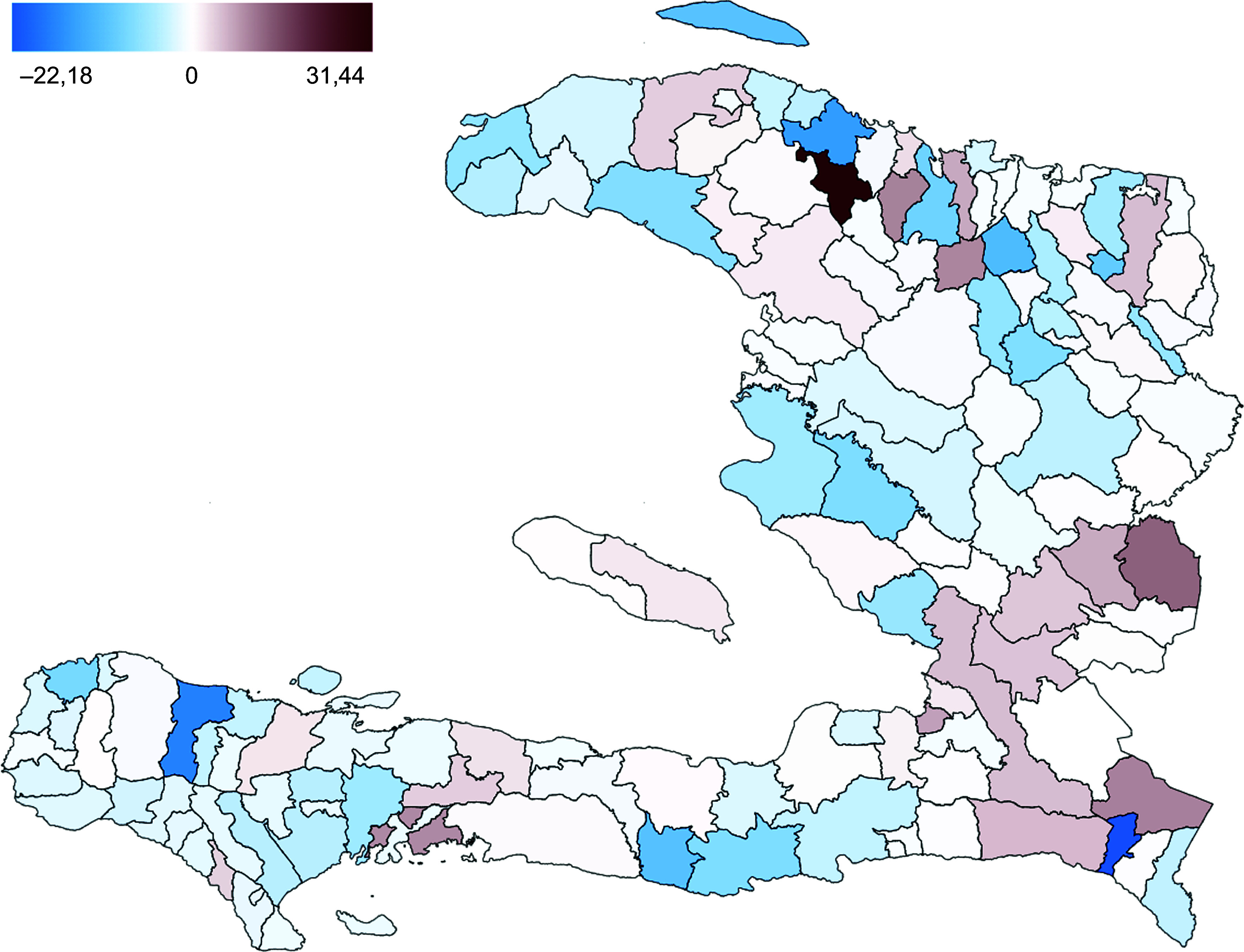
Map of commune fixed effects.

The fixed effect was tested for autocorrelation as this could affect the predictive power and lead to poor performance. In Figure 4, the fixed effect was plotted for the corresponding communes. The figure does not show clear spatial patterns of the residuals, and we conclude that spatial autocorrelation of the fixed effect is absent, in other words, that the explanatory variables are already taking up the spatially explicit impacts. The commune fixed factor would therefore, as we conjectured, reflect differences that are related to their organisation, size and specific history.

For the policy applications of the model, we imputed missing variables for education (12), average height mother (12), toilet facility (31 missing), exclusive breast-feeding (39) and error term (54) as explained in ‘Statistical analysis’ section.

### Simulation of policy interventions and policy implications

The estimated model is used to specify scenarios as packages of exogenous variables (Table 4). Each package stands for a set of interventions that aim to reduce the stunting rate. Two options are studied:

- Raising attainment of at least primary education by mothers; this can be implemented through awareness campaigns, community-based learning programmes and stimulating girls to finish their school.
- Improving WASH conditions, by ensuring access to and use of improved sanitation facilities.

**Table 4.** Policy scenarios. Baseline and targeted objectives for maternal education and sanitation facility (in percentage)

	Maternal and child conditions				WASH conditions	
Scenario	Maternal education		Exclusive breastfeeding	Average height mother	Sanitation facility	
	Baseline	Target			Baseline	Target
SanitationAccess					25·5	50·0
MaternalEducation	81·0	90·0				
LimitedIntervention	81·0	85·0			25·5	35·0
ModerateIntervention	81·0	90·0			25·5	50·0
FullIntervention	81·0	95·0			25·5	75·0

The impact of these packages is discussed individually to demonstrate their marginal contribution to reduce stunting and as a development package with combined interventions. Two variables are used for control only since these are outcome variables rather than policy ones (maternal height) or have a complex relation with stunting (exclusive breast-feeding without specifying period).

SanitationAccess concerns WASH conditions, where improved sanitation facility is aimed to reach 50 % of the population. MaternalEducation refers to maternal and child conditions, where maternal education is aimed to reach 90 % of the mothers.

**Figure 5.**
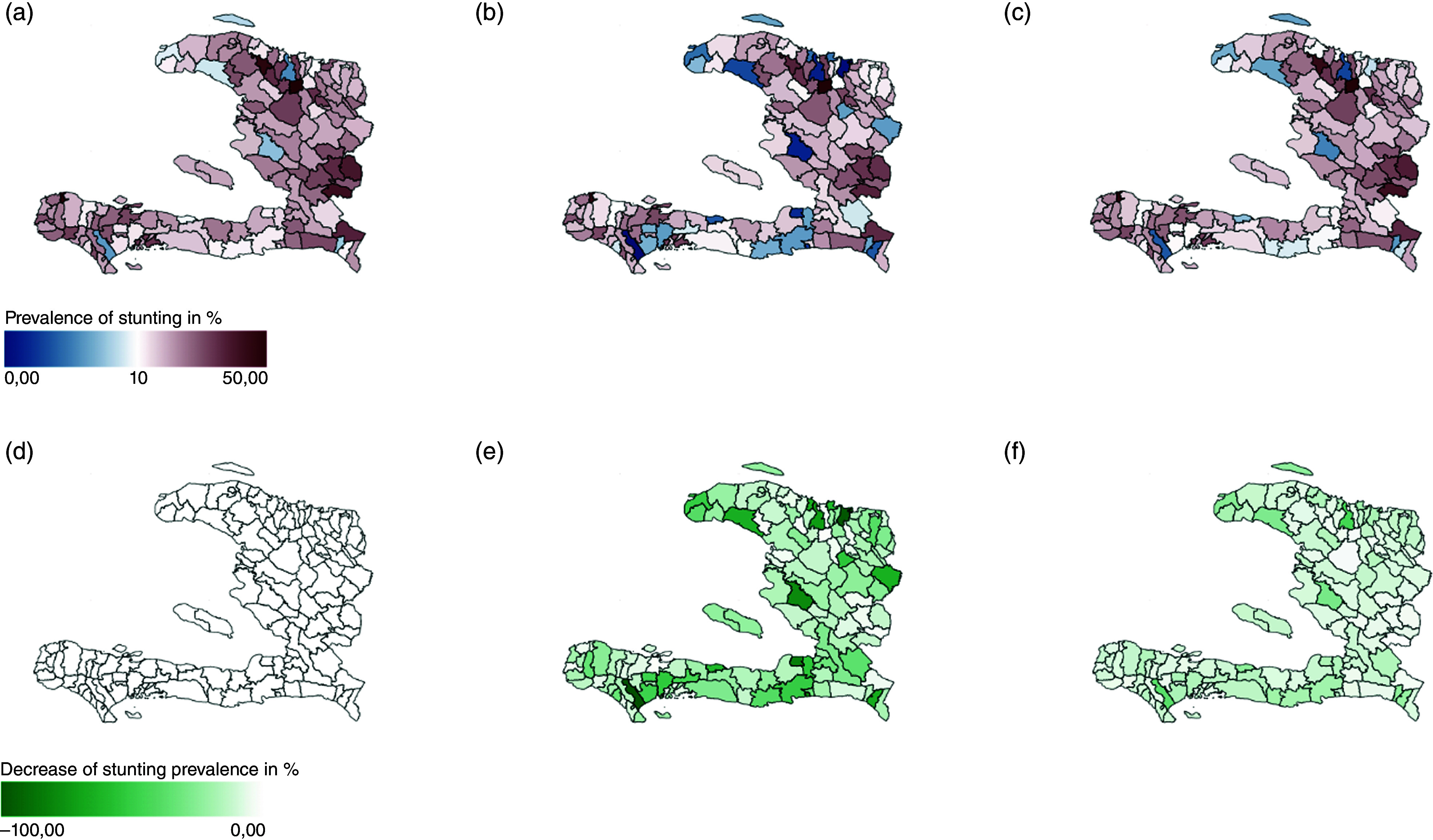
The upper row shows prevalence of stunting, the lower row the relative impact of intervention defined as difference between baseline and model outcome divided by baseline values, for scenarios: current situation (a) and (d), SanitationAccess (b) and (e) and MaternalEducation (c) and (f).

Given the need for targeted local interventions, Figure 5 shows the spatial distribution of the impact on stunting prevalence for the two single interventions. Here, the upper row shows prevalence of stunted children, the lower row shows the relative impact of the intervention. Results are shown for the current situation (a and d) and scenarios SanitationAccess (b and e) and MaternalEducation (c and f ). At national level, stunting in Haiti will be reduced by 4 % for SanitationAccess and 2 % for MaternalEducation. Increasing access to improved sanitation facilities reduces stunting rates in all communes in Haiti, while the impact of increased maternal education has a comparatively smaller effect. Expanding improved sanitation facilities nationwide is a relatively easy measure to implement with the potential for a significant reduction in stunting rates.

The second series of interventions show scenarios for different implementation levels that combine the single components of the first round. In the ‘LimitedIntervention’ implementation scenario, maternal education is targeted to reach 85 %, while access to sanitation facilities is set at 35 %. The ‘ModerateIntervention’ scenario aims to achieve 90 % maternal education coverage and 50 % improved sanitation access. Finally, the ‘FullIntervention’ implementation scenario targets 95 % maternal education coverage and 75 % improved sanitation access.

**Figure 6.**
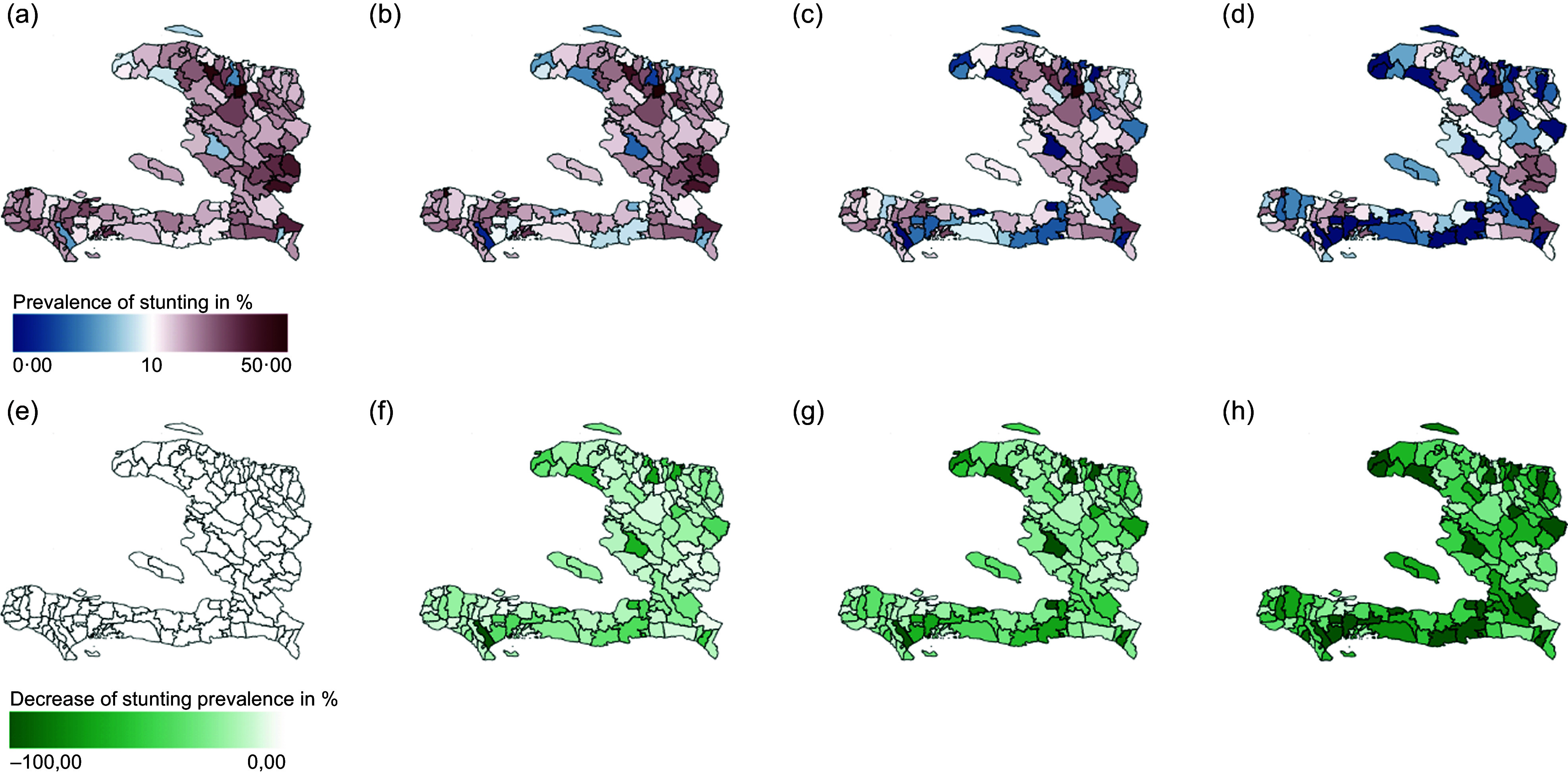
The upper row shows prevalence of stunting, the lower row the relative impact of intervention defined as difference between baseline and model outcome divided by baseline values, for scenarios: Baseline, (a) and (e), LimitedIntervention (b) and (f), ModerateIntervention (c) and (g) and FullIntervention (d) and (h).

Figure 6 shows the spatial distribution of the packaged scenarios. Analogue to the presentation of results in Figure 5, the maps of the upper row show prevalence of stunted children, and the lower row shows the relative impact of the intervention. Results are shown for the current situation (a and e) and scenarios LimitedIntervention (b and f), ModerateIntervention (c and g) and FullIntervention (d and h). At national level, stunting is expected to decrease by 2·5 %, 6 % and 10 %, regarding the scenarios LimitedIntervention, ModerateIntervention and FullIntervention, respectively.

With the implementation of the LimitedIntervention scenario, all communes are better off, but only twenty-two of 134 communes achieve a stunting prevalence below 10 %. The FullIntervention scenario results in approximately half of the communes achieving stunting rates of less than 10 %, with more than a quarter of communes showing stunting rates close to zero. The commune of Dondon stands out as its stunting rate decreases by only 7 % even with FullIntervention implementation of all interventions. In contrast, communes such as Torbeck and Acul du Nord achieve stunting rate reductions of 50 % or more.

## Discussion

This study developed a spatial decision support tool for policymakers to evaluate the effect of various interventions on childhood stunting. The tool was based on an estimated spatially explicit model with explanatory variables related to three clusters: Maternal and Child conditions, WASH conditions and Food and nutrition security and agricultural aspects. Important explanatory factors for predicting stunting prevalence are ‘sanitation facility’, ‘education of the mother’, ‘exclusive breastfeeding’ and ‘height of the mother’.

Notably, most factors in the model belong to the Maternal and Child conditions cluster. However, height of the mother is not a policy variable and was therefore held constant and not manipulated in the scenarios. Despite the time-consuming and costly nature of increasing maternal education through an intervention, this factor was included in the scenarios because of its large marginal contribution to explain stunting incidence.

Based on the univariate analysis of DHS data, Haiti is not performing well on WASH conditions and Food and Nutrition Security factors. Contrary to studies,^(40,46,47)^ which could not detect a relationship between WASH and stunting rates, we found that increasing the access to improved sanitation facilities will decrease stunting immensely and is relatively easy to implement. The spatial decision support tool could inform policymakers where their interventions can be piloted in the communes most efficiently.

In terms of impact, long-term malnutrition, as indicated by stunting, leads to the loss of productive life years and related negative impact for the economy. For an economic interpretation of the stunting results, we use the Galasso & Wagstaff^(6)^ study who quantified economic costs and benefits of policy interventions that aim to reduce the stunting rates. Concerning the costs they stipulate that in today’s workforce an average GDP per capita of 8 % in Haiti is lost due to childhood stunting. Table 5 shows income lost by childhood stunting for all scenarios projected to GDP World Bank data and UNDESA population data for 2017. In the base case scenario, no intervention results in a loss of USD 171 million (close to the USD 211 million estimated by Wong & Radin^(48)^). Benefits of interventions are substantial and can reduce economic losses by almost 50 % to 91·5 million. The costs of the interventions that lead to the reduced stunting prevalence of the aforementioned scenarios are very difficult to estimate, particularly given the uncertain situation in many parts of Haiti. For an illustration of the economic consequences of stunting reduction, we again use Galasso & Wagstaff^(6)^ who concluded that a 20 % reduction in the prevalence of stunting will cost Haiti roughly 29·5 million dollar per year. Positive economic benefits will only begin to emerge 15 years after program implementation when the initial cohort enters the workforce. At that time, a total of 442·5 million dollars will have been spent to reduce stunting prevalence and the first group who benefitted from the program will start earning 55·9 million per year. Using a cash flow analysis, we observe that it requires another 8 years following the entrance of 15-year-olds into the workforce for the benefits to outweigh the costs (break-even). Although we reiterate that for an effective and efficient implementation of this plan, a stable political situation and good governance are required, we also stress the fact that our analysis provides guidelines for local-level interventions with associated benefits, enabling a ripple effect that extends across larger parts of the country.

**Table 5.** Model scenarios with stunting rates among children under five and projected income loss

Model	Stunting percentage	Income lost by stunting (million dollars)
Without interference	21·5	171·0
SanitationAccess	17·6	140·0
MaternalEducation	19·6	155·9
LimitedIntervention	19·1	151·9
ModerateIntervention	15·7	124·9
FullIntervention	11·5	91·5

Reducing stunting in Haiti is not only important from a position of public health but also generates positive economic effects. Without interference, Haiti’s GDP loses 171 million USD due to childhood stunting, whereas even with a LimitedIntervention implementation, the country can reduce this loss by 20 million USD over time. These economic benefits can only be realised with long-term perseverance that goes beyond political election cycles.

Studies^(49,50)^ emphasised that investing in nutritional programmes is among the best strategies for countries aiming to enhance and maintain their wealth. A review of McGovern *et al.* ^(50)^ suggests that reduction of stunting requires a comprehensive approach that emphasises quality of economic growth, intermediary factors such as sanitation, education, quality of diets, access to basic health services and a combination of poverty reduction strategies and direct nutrition interventions. However, our results show that focussing on WASH alone already can bring substantial improvements, providing a ‘hierarchy’ in urgency of policies to be implemented. Still, future research should focus on cost–benefit analysis of broader intervention packages than the ones considered in this paper. We emphasise that the flexible approach utilised in this study can easily be adapted to study broader packages, but also other countries. The basic requirements for having spatially explicit explanatory variables and health statistics at the district level are straightforward to fulfil. Additionally, modelling and visualisation can be accomplished using freely available statistical and GIS software.

The study obviously also has limitations. First and foremost, we are fully aware of the potential impact of the widespread lawlessness and ongoing armed conflicts on the accessibility and operational feasibility of food programmes. Yet, we remain hopeful that the current international peacekeeping forces in Haiti and negotiations between armed groups and government will restore political and economic stability to enable the execution of targeted food policies. Second, the data from 2016 to 2017, published in 2018, are approximately seven to nine years old and, possibly, do not capture latest developments. Yet, since the devastating 2010 earthquake, Haiti endured a prolonged period of economic stagnation with limited external disturbances and available evidence suggests that the food security situation remained relatively stable over the past decade. For example, between 2014 and 2022, prevalence of child stunting in Haiti remained largely unchanged, at 22·7 % in 2014 and 21·9 % in 2022, while undernourishment affected 42·6 % of the population in 2014 and 47·2 % in 2022^(4)^. A third key limitation is data quality, particularly the presence of missing values across most variables, which can reduce model performance. To address this, missing data points were imputed using Euclidean distance based on Tobler’s First Law of Geography. Finally, as yet, the scenarios cover only three main directions. Nonetheless, the model is sufficiently flexible to evaluate other scenarios where interventions at different levels can be tested.

To conclude, this study has a robust multidisciplinary character that makes it well-suited to analyse the food security conditions in Haiti. The estimated model that relates undernutrition to socio-economic conditions and biophysical factors is fully operationalised into a decision support tool that informs policymakers on effectiveness of their interventions. Additionally, the spatially explicit component of the decision support tool helps to prioritise areas where aid is most urgently needed. With the introduction of district fixed effects, location-specific confounding effects are addressed as well.

Food security in Haiti requires short-term aid on par with long-term strategies to address the root causes of instability and poverty. Stable governance and long-term investments to empower farmers in their productive capacity are crucial to achieve a more food secure future in Haiti. Given the prevailing unrest and political instability, a strategy starting with relatively safe communes and gradually scaling to other, more unstable communes could be a viable strategy to start.
